# Mobile assisted language learning in learning English through social networking tools: An account of Instagram feed-based tasks on learning grammar and attitude among English as a foreign language learners

**DOI:** 10.3389/fpsyg.2022.1012004

**Published:** 2022-09-15

**Authors:** Chunyan Teng, Tahereh Heydarnejad, Md. Kamrul Hasan, Abdulfattah Omar, Leeda Sarabani

**Affiliations:** ^1^Hangzhou College of Preschool Teacher Education, Zhejiang Normal University, Jinhua, China; ^2^Department of English Language, Faculty of Literature and Humanities, University of Gonabad, Gonabad, Iran; ^3^English Language Institute, School of Humanities and Social Sciences, United International University, Dhaka, Bangladesh; ^4^Department of English, College of Science and Humanities, Prince Sattam Bin Abdulaziz University, Al-Kharj, Saudi Arabia; ^5^Department of English, Faculty of Arts, Port Said University, Port Said, Egypt; ^6^Department of English, Kabul University, Kabul, Afghanistan

**Keywords:** Instagram feed-based tasks, game-based learning, learning grammar, EFL learners, MALL (mobile assisted language learning)

## Abstract

Advancement of social media in the modern era provides a good incentive for researchers to unleash the potential of social networking (SN) tools in order to improve education. Despite the significant role of social media in affecting second/foreign language (L2) learning processes, few empirical studies have tried to find out how Instagram feed-based tasks affect learning grammar structure. To fill this lacuna of research, the current study set forth to delve into the influence of Instagram feed-based tasks on learning grammar among English as a foreign language (EFL) learners. In so doing, a sample of 84 intermediate EFL learners were randomly divided into experimental and control groups. The learners in the control group received regular online instruction via webinar platforms. In contrast, the learners in the experimental group were exposed to Instagram feed-based tasks. Data inspection applying one-way ANCOVA indicated that the learners in the experimental group outperformed their counterparts in the control group. The results highlighted the significant contributions of Instagram feed-based tasks in fostering learning grammar. Furthermore, EFL learners’ positive attitudes toward using Instagram Feed-based Tasks in Learning Grammar was concluded. The implications of this study may redound to the benefits of language learners, teachers, curriculum designers, as well as policy makers in providing opportunities for further practice of Instagram feed-based tasks in language learning and teaching.

## Introduction

Rapid development in technology calls for more innovative teaching and learning techniques. In the past half-century, the research on Social Networking (SN) has blossomed, particularly during the COVID-19 Pandemic Crisis, leading to the near-total closures of schools and minimal relationships among people. The COVID-19 pandemic has disrupted education in many countries; thus, as an emergent response, many countries focused on implementing remote learning modalities. Meaningful two-way interaction between students and their teachers during COVID-19 pandemic was mostly solved via online programs and social media (Shadiev and Yang, 2020; Kamasak et al., 2021; Li et al., 2021; Lei et al., 2022). Aside online programming, Mobile-Assisted Language Learning (MALL) in learning English through SN tools excited special attention in recent years.

Social media language learning is a method of language acquisition that uses socially constructed Web 2.0 platforms to facilitate learning of the target language. SN tools as computer aided web-based tools, support communication, collaboration, and information sharing. SN through MALL-oriented applications opens new perspectives in language learning (Navarro-pablo et al., 2019; Woods, 2020; Liu et al., 2021). The concept of MALL can be defined as a mixture of digital technology with language learning (Crompton and Burke, 2018; Ahmadi and Tabatabaei, 2021; Kamasak et al., 2021; Lei et al., 2022). MALL has evolved to support Students’ language learning through activities to be performed on mobile devices at anytime and anywhere (Gonulal, 2019; Wrigglesworth, 2019; Zai, 2021). During the Covid 19 Pandemic, social media complement the teaching-learning process and created an avenue for sharing educational contents, interaction, and communication.

In line with this flourishing interest in the usage of social media, different studies have been conducted to demonstrate the influence of social media platforms on education, especially in the field of foreign language teaching and learning (e.g., Krutka and Carpenter, 2016; Taskiran et al., 2016; Vivakaran and Neelamalar, 2018; Maulina et al., 2021). Among social media platforms, Instagram has attracted great attention in recent years (e.g., Erarslan, 2019; Gonulal, 2019; Lailiyah and Setiyaningsih, 2020). Instagram bestowed online communities for sharing knowledge and promoting learning. It can offer innovative ways of learning, which is part of the interest of this generation of learners, as digital natives (Erarslan, 2019). Instagram can be used as a source of a number of activities in language classrooms such as digital storytelling, grammar activities through photos, role plays, reading, speaking activities through videos (Handayani, 2016; Devana and Afifah, 2021). Soviyah and Etikaningsih (2018) asserted that Instagram can influence learners’ autonomy, motivation, self-assessment, group working, and academic achievement. Although Instagram is one of the most popular social media platforms, its efficiency for language learning purposes, in particular learning grammar has not been studied in detail, and the review of the existing literature implied that there is ample room for future studies. Keeping these standpoints in mind, this study sought to shed more light on the realm of technology-based instruction by exploring the possible influence of Instagram feed-based tasks on learning grammar among EFL university learners. Furthermore, EFL university learners’ attitudes toward learning grammar via Instagram feed-based tasks was the target of this research. Aiming to fill the existing gap in the literature, this study seeks to answer the following research question:

RQ1. Do Instagram Feed-based Tasks influence Learning Grammar among EFL Learners?

RQ2. What are EFL learners’ perceptions toward using Instagram Feed-based Tasks in Learning Grammar?

## Literature review

The relevant literature on social media, MALL, and Instagram, as well as their efficacy in education, are reviewed in this section.

### Social media and education

The emergence of the Internet opens a new horizon for technologies of communication and information sharing. This situation was the triggering step for the birth of social media. The need for an affordable, portable, and accessible information and communication technology (ICT) tool gave birth to social media. According to Manning (2014), two ages are considered for the development of media: the broadcast age and the interactive age. The broadcast age focused on one entity (e.g., a radio or television station and newspaper company) designed to distribute messages to many people. They possess indirect, delayed, and impersonal feedback. Furthermore, the mediated communication between individuals is limited. Contrarily, social media refer to new forms of media that evolve around interactivity. Prompt and direct feedback is also possible through social media (Manning, 2014; Vadivel et al., 2021).

According to Manca (2020), social media is internet-based applications for sharing images (e.g., Instagram), information organization (e.g., Pinterest), photo or video messaging (e.g., Skype), instant messaging (e.g., WhatsApp), or a combination of all (e.g., Facebook). From a similar perspective, Grahl (2013) divided social media into six different but overlapping categories as follows: (1) social networks (e.g., Facebook, LinkedIn); (2) bookmarking sites (e.g., Delicious, StumbleUpon); (3) social news (e.g., Digg, Reddit); (4) media sharing (e.g., Instagram, YouTube, Flickr); (5) microblogging (e.g., Twitter); and (6) blogging, particularly comments, and forums. The affordability, portability, and accessibility of social media made is widespread around the world very soon. Their massive popularity has attracted the attention of many educators to investigate their pedagogical affordances, and language instruction was not an exception.

Social media platforms revolutionized language teaching and learning; they provided a socially engaging learning atmosphere (Vadivel and Beena, 2019; Manca, 2020; Ahmadi and Tabatabaei, 2021; Rasyiid et al., 2021). More importantly, social media platforms are flexible; that is, they can be used across all educational settings (e.g., K-12 and higher education) as well as formal and informal learning contexts (e.g., Barrot, 2021). The two dimensions of formality and informality of social media and how to make a distinction between them are in question among educationalists. Some argue that these two dimensions are in a continuum (e.g., Sefton-Green, 2004) contrarily others consider them binary (e.g., Eshach, 2007). In a recent attempt, Manca and Ranieri (2016) as well as Greenhow and Lewin (2016), discussed that this distinction should be conceived as interconnected and as a continuum.

Adoption of social media in the educational context is theoretically supported by Technology Acceptance Model (TAM), which highlights the external variables that could foster the appliance of technology (e.g., social media), which in consequence, influences the learners’ attitudes toward learning (Davis, 1989; Namaziandost et al., 2021). A plethora of studies has shed light on the contributions of social media to Students’ wellbeing. For instance, Dizon (2016) conducted a quasi-experimental study to investigate the usefulness of Facebook in fostering the writing fluency of EFL learners. The ultimate findings of this study confirmed the efficacy of Facebook in promoting second language writing. In a mixed-method study, Students’ perceptions of WeChat as a language learning tool were explored at a Chinese university by Zou et al. (2018). Based on their outcomes, the integration of WeChat into language instruction leads to the learners’ progress. In the same line of inquiry, Lenkaitis (2020) employed Zoom (videoconferencing platform) to explore its efficacy in language learning and learner autonomy. This study provides evidence of the effectiveness of videoconferencing activities with tools like Zoom in fostering language learning and increasing learners’ autonomy.

### Mobile assisted language learning and education

Over recent years, mobile device technology has been developed very quickly. Besides oral communication, which is the main aim of designing the mobile phones, recent changes in mobile device technology define various functions for them. With the use of the internet, the users are enabled to access the internet to search for information, email, read e-books, and shop irrespective of time and place (Pachler et al., 2010). The integration of mobile device technology into teaching and learning was introduced as mobile assisted language learning (MALL), which is defined as a specialization of mobile learning (m-Learning) and a branch of computer-assisted language learning (CALL) (Kukulska-Hulme and Shield, 2008; Li et al., 2020; Namaziandost et al., 2020). According to Huang et al. (2012), mobile technologies have many positive advantages: flexibility, low cost, small size, and user-friendliness, to name a few.

Despite the rapid evolution of MALL, no agreed-upon definition exists for this concept. As Palalas (2011) viewed m-learning as an active process using mobile technologies that empower students to access, create, and share knowledge together. From a similar perspective, m-learning is defined as learning across locations and contexts with the help of mobile technologies (Crompton, 2014). Moreover, no direct and field-specific theory was raised to capture MALL. The existing theories were borrowed from other fields. For example, Applied Cognitive Load Theory (Oberg and Daniels, 2013), and Dual-Coding Theory (Delprato, 2009) were originated from the cognitive psychology and TAM (Cheng et al., 2010) was formulated based on informatics research. The Theory of m-Learning postulated by Sharples et al. (2007) could demonstrate and justify the applicability of m-Learning across locations, times, topics, and technologies.

Several studies were conducted to touch on the issue of MALL from different perspectives and other contexts. For example, Chen (2013) explored the influence of employing a Tablet PC for informal learning of English outside of the classroom setting. The results of this study revealed that a Tablet PC could foster the learning of English a great deal. In another study, Chien (2016) demonstrated that m-learning raises self-dependent and self-motivated learners. Similarly, Zou et al. (2020) concluded that collaborative approaches in m-Learning enhance learning. Considering vocabulary learning, the study of Zhang and Pérez-Paredes (2021) was evidence of using vocabulary learning applications and improving vocabulary acquisition. In the same vein, Chen and Jia (2020) concluded that MALL enhances learning because they are designed based on learners’ need analysis and pedagogic goals.

### Instagram and education

The appearance of Instagram dates back to 2010 with photo sharing platform, and over the years, other enhancements such as texting, video, and story sharing has been added (Ellison, 2017). Instagram is a mobile application that provides users the opportunity to take instant snapshots of images, current activities, upload pictures, text messaging, and posting videos (Chen, 2021). As Salomon (2013) argues, Instagram is a type of digital literacy enhancing the teaching and learning process. Instagram proposes different contextualized visual information, socially associated network of learners, as well as comment and tagging feature (Chen, 2021) to be directed and used in language teaching and learning. Furthermore, Shazali et al. (2019) expressed that learning time can be expanded productively via suggested activities on Instagram. The mobility and accessibility of Instagram make dynamic language exposure (Al-Ali, 2014), which is necessary for successful language learning.

Recent approaches to teaching language skills reported successful improvement of the learners as well as increasing learners’ motivations to learn due to the creative and fascinating learning atmosphere that Instagram provides (Zheng et al., 2018; Pujiati et al., 2019; Min and Hashim, 2022). For improving speaking skills, Instagram can offer short videos like tutorial videos or long videos like a live broadcasts and public speaking (Devana and Afifah, 2021). Considering writing skills, Instagram suggests communicative purposes and long-distance service (Soviyah and Etikaningsih, 2018). Learners can text messages privately or publicly as part of their designed class activity to improve their writing skills (Rasyiid et al., 2021). Furthermore, Instagram is a suitable source for practicing grammar and vocabulary due to its nature in providing materials with images and videos (Rasyiid et al., 2021).

### Theoretical framework

Providing the psychological comfort of the learners is one of the major objectives of a successful education system. The engaging learning procedures and interactive tools in the educational process can stimulate learners to act effectively. To capture the learning processes, Skehan (1998) proposed a model of learning called PPP (presentation, practice, and production) that attempts to introduce three steps of learning a language until reaching fluency. Criado (2013) referred to this model as the mainstream of English as a foreign language (EFL) style. Based on Anderson (2017), PPP model was introduced at the dawn of CLT. PPP model suggests that individual language items should be explicitly taught (P1), then practiced (P2) in isolated sentences through controlled activities, and at the final stage, learners produce (P3) these items in a freer manner (Harmer, 2007). Theoretically, PPP model is supported by skill acquisition theory (SAT) which emphasizes that learning triggers with explicit attention to a linguistic feature to establish declarative knowledge which is then proceduralized and automatized through practice (DeKeyser, 2007). Some previous studies witnessed the effectiveness of implementing PPP in explicit instruction (Norris and Ortega, 2000; Spada and Tomita, 2010; Anderson, 2017). Considering three steps of learning a language (PPP), the researchers of this study attempted to complete the first and second steps (i.e., presentation and practice) by using Instagram feed-based tasks instead of traditional drilling to inspect any possible changes in the production stage of the learners.

Furthermore, this study is fundamentally based on connectivism. This new learning theory (connectivism) argues that students should combine thoughts, theories, and general information in a useful manner. It accepts that technology is a major part of the learning process and that learners’ constant connectedness provides opportunities to make choices about their learning. Connectivism originated from distributed learning (Siemens, 2004) and described the digital forms of communication within the course. In other words, the acquisition of skills through digital connections and social media are defined and supported by connectivism (Greenhow and Lewin, 2016). The concept of connectivism has implications in self-organization, which implicitly emphasizes the role of self-organization of the learners who create the larger self-organizing knowledge structures (Greenhow and Lewin, 2016).

## Materials and methods

The current study is quantitative in nature and uses a pre-test-post-test quasi-experimental design. In the following, the undertaken steps are introduced in detail.

### Participants

Among 125 freshmen EFL learners studying at the University of Gonabad, northeast of Iran, a sample of 84 participants (50 females, 34 males) was chosen based on the results of the Oxford Quick Placement Test. The results of the Oxford Quick Placement Test indicated that the participants were at the intermediate level of English language proficiency. Also, during the research project, they did not attend extra English classes. Therefore, the level of English language proficiency of the participants was similar at the beginning of the study. They were from diverse socio-economic backgrounds, with the age range from 18 to 22. There were 43 students (28 female and 15 male) in the experimental group and 41 students (22 female and 19 male) in the control group. According to their syllabi, the students had to take an English grammar course in the first semester of the academic year (16 sessions). The students were fully aware of the voluntary nature of the study and gave informed consent to participate in this research project.

### Instruments

The following instruments were utilized in this research:

#### Oxford quick placement test

To determine the Students’ level of English language proficiency, the Oxford Quick Placement Test was administered. The score range in the Oxford Quick Placement Test is 0.1–0.9, and the scores between 0.4 and 0.6 are determined as an intermediate level of English language proficiency. The reliability of the Oxford Quick Placement Test in this study was 0.91.

#### English grammar pre-test and post-test

A researcher-made test was designed based on the topics of the suggested materials (Understanding and Using English Grammar, Azar and Hagen, 2009). This test contains 40 items in the form of multiple choice (15 items), fill in the blanks (15 items), and correct the errors (10 items). To inspect the face and content validity of the items, expert judgment was employed. In so doing, two psychometricians and six EFL professors were invited to evaluate the quality of the items. Based on their comments, some items were revised. Following this step, this test was used for a sample of 34 university students similar to the target population to check the test-retest reliability. To gauge the stability of the results over time, the same test was re-administered to the same participant after 2 months. Based on the results of the Pearson correlation coefficients, a high test-retest reliability of the test was confirmed (*r* = 0.90, *p* < 0.05).

#### Attitudes toward using social media for learning purposes

To investigate the learners’ attitudes toward using social media for learning purposes, an instrument developed and validated by Shaheen et al. (2020) was utilized. This instrument involves 19 items with five sections as following: (a) demographic information, (b) the learners’ experience with social media platforms (one item), (c) social media usage (five items), (d) attitudes toward applying social media to enhance learning process (six items), and (e) the advantages of social media for learning (one item). This instrument is designed in multiple-choice items, five-point Likert scales, and free text. The reliability of this instrument estimated via Cronbach’s alpha was acceptable (ranging from 0.759 to 0.878).

### Procedure

In the first phase, the current study used a quasi-experimental design, and the participants were assigned to groups based on non-random criteria. In the beginning, the Oxford Quick Placement Test was used to determine the Students’ level of English language proficiency. Based on the results of the Oxford Quick Placement Test (score range 0.1–0.9) and considering the cut score (0.4–0.6), 84 students were chosen for the present study. The students with the higher scores (between 0.7 and 0.9), showing high language proficiency were omitted, and the students with and intermediate level of English language proficiency were asked to participate in this study. Furthermore, the selected participants were asked not to attend any extra English classes during this research project. Prior to administering the treatment, a pre-test was carried out.

After the pre-test, the instruction was done by one of the researchers, who was the instructor of all of the courses in both the experimental and control groups. This study was carried out during one semester (16 sessions) in 2021. As part of their schedule, freshmen EFL learners were supposed to pass English grammar. To practice English grammar, Understanding and Using English Grammar (Azar and Hagen, 2009) was utilized for both groups. The students in the control group (41 students) received regular online instruction via webinar platforms (Adobe connect). In contrast, learners in the experimental group (43 students) practice English grammar via a social page of Instagram designed by the researchers. This page introduced various tasks intended to teach and practice English grammar based on the intended book (Understanding and Using English Grammar, Azar and Hagen, 2009) and the class syllabus. Each topic of this book was introduced with the help of interesting posts containing images, audio, and films. Both teachers and learners are capable of sending images, audios, and videos via Instagram. Moreover, the learners can ask and answer questions and receive feedback. To avoid any inconsistency during the treatment, the learners in the experimental group were asked not to share the information with their counterparts in the control group.

At the end of the semester (16 sessions), after the instruction was completed, a post-test was conducted to inspect the learners’ achievements in both the control and experimental groups and investigate the effectiveness of the program. Both pre-test and post-test were scored by 3 EFL teachers to ensure the reliability of the scores. The average score of pre- and post-test for each student was finally considered. Furthermore, to enrich the data, the learners in the experimental group were asked to complete the questionnaire of attitudes toward using social media (Shaheen et al., 2020). It was conducted via a web-based platform. The language of the questionnaire was English since all the participants were qualified to understand English. Conducting the electronic survey enables researchers to collect data from different regions.

### Data analysis

To explore the efficiency of Instagram Feed-based Tasks on Learning Grammar among EFL Learners, one-way ANCOVA was run. In addition, a descriptive analysis of the questionnaire was employed to answer the second research question.

## Results

### Results for the first research question

To analyze the data in the first phase, the Statistical Package for Social Sciences (IBM SPSS, version 26) was used. The descriptive statistics for the pre-test and post-test of both the control and experimental groups are presented in Table 1.

**TABLE 1:** Descriptive statistics for pre-test and post-test of the control and experimental groups.

Groups		Pre-test grammar	Post-test grammar
Control	N	41	41
	Mean	2.835	2.882
	Std. Deviation	0.599	0.607
	Variance	0.359	0.368
	Minimum	1.900	2.000
	Maximum	4.000	4.000
Experimental	N	43	43
	Mean	2.955	3.509
	Std. Deviation	0.652	0.419
	Variance	0.425	0.176
	Minimum	2.167	2.767
	Maximum	4.000	4.000

The reported data in Table 1 indicates that the range of scores in pre and post-test grammar for the control group are as follows: 1.900–4.000 and 2.000–4.000, respectively. For the experimental group, the range of the scores in pre and post-test are as follows: 2.167–4.000 and 2.767–4.000, respectively. Thus, it can be inferred that applying a serious game for teaching grammar enhanced English grammar learning in the experimental group. Following this step, the normality of the data was explored via the Kolmogorov-Smirnov test.

The results of the Kolmogorov-Smirnov test indicated that the distribution of scores was normal (Table 2). Thus, the parametric test of the independent samples *t*-test was utilized to inspect whether the differences between the two groups were statistically significant (Table 3).

**TABLE 2:** The Kolmogorov-Smirnov test for checking the normality of scores.

Groups		Grammar. pre	Grammar. post
Control	Kolmogorov-Smirnov Z	0.666	0.694
	Asymp. Sig. (2-tailed)	0.767	0.721
Experimental	Kolmogorov-Smirnov Z	0.806	0.826
	Asymp. Sig. (2-tailed)	0.534	0.502

**TABLE 3:** *T*-test for the main four skills in pre- and post-test.

		Levene’s test for equality of variances		*t*-test for equality of means				
		F	Sig.	T	df	Sig. (2-tailed)	95% Confidence interval of the difference	
							Lower	Upper
Grammar. pre	Equal variances assumed	0.196	0.661	−0.625	41	0.536	−0.508	0.268
	Equal variances not assumed			−0.628	40.863	0.533	−0.505	0.265
Grammar. post	Equal variances assumed	2.046	0.160	−3.985	41	0.0003	−0.945	−0.309
	Equal variances not assumed			−3.885	33.096	0.0005	−0.955	−0.299

Based on Table 3, the level of significance for the variance of the control and experimental groups in the pre-test of grammar was 0.661 (*p* > 0.05), therefore the variance of the two groups was equal. Regarding the means of the two groups in the pre-test of grammar, no significant difference between the control and experimental group was indicated. Considering the post-test of speaking, the level of significance for the variance of the two groups was (0.160, *p* > 0.05). Moreover, a significant difference between control and experimental groups was obtained in the post-test of grammar as the level of significance for the means of the two groups was statistically significant.

### Results for the second research question

#### Students’ demographic characteristics

On account of the design of the electronic survey (each part in the electronic survey form was designed to be necessarily linked), no data were missed. Their age range was from 18 to 21 years (22 female and 19 male). Based on their responses, all of them have smart devices, and 90.2% have computers at home.

#### Experience with social media platforms

About the experience with social media platforms, the answers were different. About 85.3% of the participants in the experimental group had the best experiences with Instagram. The other social media platforms were endorsed by students were as following: WhatsApp (77.9%), YouTube (59.6%), and Twitter (47.4%), respectively.

#### Uses of social media

As the data analysis revealed, 16 males and 20 females always use social media platforms. Among the social media platforms, Instagram was the most and Wikis the least preferred social media platform on a daily basis among the participants in the experimental group. Moreover, the findings showed that males mostly preferred WhatsApp and females preferred Instagram. Nearly all the participants (36), 16 males (92.1%) and 20 females (95.3%), stated that they “always,” “often,” and “sometimes” employ social media for learning objectives. Based on the outcomes, almost all the participants spend 2–3 h in a typical day utilizing social media and social networks.

#### Attitudes toward using social media to support learning

As Table 4 summarized, the overall attitudes of students toward applying social media for learning purposes were positive. Considering item number one, 88% of them strongly agreed that social media were important because they helped them with learning support. About 83.8% of the students, strongly agreed that it was a good idea to use social media to support learning. Regarding item number three, 85.8% of the students strongly agreed that learning online through using social media was fun. 83.7% found using social media for learning as a very desirable tool for learning. Moreover, 81.2% of the students reported that communicating with their classmates and instructor using social media provides them with good learning experiences. Considering item number five, 81.2% of the students preferred to join classmates in collaborative projects using social media. Among the students, 85.2% preferred to attend a class where the instructors applying social media in their teaching. Furthermore, the findings revealed that 39.1% of the students found it difficult to stop social media, when they started using it to support learning.

**TABLE 4:** Students’ attitude toward using social media to support learning.

			SD	D	N	A	SA
1	Social media are important because they help me with learning support.	Male	5.6	7.1	19.3	23.3	43.7
		Female	4.2	8.5	20.3	22.7	44.3
		Mean	4.9	7.8	19.8	23.0	44.0
2	In my opinion, using social media to support learning is a good idea.	Male	4.5	15.1	19.4	26.5	34.5
		Female	3.8	8.6	14.2	24.1	49.3
		Mean	4.1	11.8	16.8	25.3	41.9
3	I find learning online through using social media is fun.	Male	11.5	5.3	11.2	29.4	42.6
		Female	6.1	8.6	14.5	31.6	43.2
		Mean	8.8	7.1	12.85	30.5	42.9
4	I find using social media for learning is very desirable for me.	Male	4.1	10.7	17.2	24.6	43.4
		Female	9.4	8.4	20.6	21.3	40.3
		Mean	6.7	9.5	18.9	22.9	41.8
5	I prefer to join classmates in collaborative projects using social media.	Male	6.1	9.5	17.4	21.4	45.6
		Female	6.4	8.3	20.5	23.6	41.2
		Mean	6.2	8.9	18.9	22.5	39.9
6	Communicating with my classmates and instructors using social media provides me with good learning experiences.	Male	6.6	9.9	20.6	26.4	36.5
		Female	3.0	10.1	11.7	30.5	44.7
		Mean	4.8	10.0	16.1	28.4	40.6
7	I prefer to attend a class where the instructors using social media in his/her teaching.	Male	4.2	8.0	14.0	29.5	43.3
		Female	4.5	10.7	20.1	22.8	41.9
		Mean	4.35	9.35	18.4	26.15	40.6
8	When I started using social media to support learning, I found it difficult to stop it.	Male	11.4	7.6	25.4	35.4	20.2
		Female	6.7	19.1	41.1	14.2	18.9
		Mean	9.0	13.35	33.25	24.8	19.55

#### Benefits of social media for the learning process

Table 5 demonstrates the advantages of using social media for learning purposes from learners’ perspectives. With regard to item number one, 85.2% of the students strongly agreed that they could find many educational resources, links, programs, and topics of discussion when I used social media. Moreover, 80% of the learners strongly agreed that social media encouraged them to learn better than traditional teaching methods. About 82.3% of the students believed that their writing skills developed when they communicated with others through social media. As the data screening revealed, 92.6% of the students believed that watching videos through social media developed their grammar skills. Considering item number five, 91.4% of the learners strongly agreed that discussing and exchanging views with others using social media developed their critical thinking skills. Based on the results, 82.6% of the learners reported that communicating and interacting with their classmates and instructors through the social media helped them develop their social skills. About 82.6% of the learners strongly agreed that applications and programs provided by social media helped them to be more creative in their projects and assignments. Regarding item number eight, 81.3% of the learners strongly agreed that social media allowed them to learn collaboratively with others who have the same interests. About 82.4% of the learners, learning online through social media enhanced self-independent learning. 86.2% of the learners strongly agreed that they could learn anytime and anywhere using social media. Referring to the other contributions of social media, 90.7% of the learners believed that they could express their opinions and thoughts more freely through social media than in face-to-face discussions with their instructors and classmates in the classroom. In conclusion, 93.6% of the learners strongly agreed that the use of social media for the learning purposes develops their academic performance.

**TABLE 5:** Benefits of social media for learning process.

			SD	D	N	A	SA
1	I find many educational resources, links, programs, and topics of discussion when I use social media.	Male	8.6	11.0	20.1	21.0	39.3
		Female	3.8	7.3	19.5	23.6	45.9
		Mean	6.2	9.1	19.8	22.4	42.6
2	Social media encourage me to learn better than traditional teaching methods.	Male	8.9	13.9	12.3	25.3	39.6
		Female	6.2	10.2	11.5	31.7	40.4
		Mean	7.5	12.0	11.9	28.5	40.0
3	My writing skills develop when I communicate with others through social media.	Male	3.8	16.5	17.7	20.4	41.6
		Female	5.5	10.9	19.5	23.4	40.7
		Mean	4.65	13.7	18.6	21.9	41.1
4	Watching videos through social media develops my listening skills.	Male	5.1	11.4	15.2	25.3	43.0
		Female	2.0	10.5	14.5	23.4	49.6
		Mean	3.5	10.	14.8	24.3	39.3
5	Discussing and exchanging views with others using social media develops my critical thinking skills.	Male	3.4	10.3	15.0	28.4	42.9
		Female	3.3	6.9	17.5	23.8	48.5
		Mean	3.3	8.6	16.2	26.1	45.7
6	Communicating and interacting with my classmates and instructors through social media helps me develop my social skills.	Male	7.3	9.7	18.3	24.6	40.1
		Female	5.0	6.1	20.6	25.8	42.5
		Mean	6.15	7.9	19.45	23.0	41.3
7	Applications and programs provided by social media help me to be more creative in my projects and assignments.	Male	5.7	11.1	22.5	22.9	37.8
		Female	5.3	6.3	20.2	23.1	44.1
		Mean	5.5	8.7	21.35	25	39.45
8	Social media help me to learn collaboratively with others who have the same interests.	Male	5.0	9.4	22.6	24.3	38.7
		Female	3.2	10.8	20.1	23.3	42.6
		Mean	4.1	10.1	21.3	25.8	40.65
9	Learning online through social media enhances the self-independent learning I have.	Male	6.0	11.3	20.6	23.0	39.1
		Female	2.0	4.7	18.0	31.0	43.3
		Mean	4.0	8.0	19.3	27.0	41.2
10	I can learn anytime and anywhere using social media.	Male	6.4	10.2	15.0	27.4	41.0
		Female	3.1	5.8	14.1	31.9	45.2
		Mean	4.7	8.0	14.5	29.6	41.6
11	I express my opinions and thoughts more freely through social media than in face-to-face discussions with my instructors and classmates in the classroom.	Male	6.1	6.6	14.3	29.5	43.6
		Female	6.3	9.2	10.6	26.8	47.1
		Mean	6.2	7.9	12.45	28.15	45.35
12	In general, the use of social media for learning purposes develops my academic performance.	Male	3.8	8.9	12.8	24.4	50.2
		Female	6.8	10.3	14.3	25.1	43.4
		Mean	5.3	9.6	13.55	24.75	46.8

## Discussion

In this section, the results of the study with regard to the prior theories and findings of the previous studies were discussed:

### Discussing the first research question

This study was an attempt to depict the issue of MALL in learning English through SN tools, with the focus on English grammar learning via Instagram feed-based tasks. As the data show, Instagram feed-based tasks had a significant effect on the improvement of English learning grammar. That is, the students in the experimental group outperformed their peers in the control group. The results highlight a significant potentiality of MALL in learning English through SN tools (Instagram platform in this study), that is, teaching and learning indirectly with more enthusiasm. The outcomes of this study illuminated the contribution of Instagram feed-based tasks in enhancing the process of grammar learning.

It is worth highlighting that, Instagram feed-based tasks stimulate novelty, creativity, and engagement for the learners. More specifically, the tasks on this page were designed in a way that learners are supposed to follow all the posts step by step carefully, and all the facts and data were necessary for being clear about the next step. This opportunity may increase the possibility of long-term retention of grammatical structures and a satisfactory rehearsal of the rules. This finding is confirmed by Naderi and Akrami (2018) found out that MALL improves learners’ vocabulary retention. The results of Kurt and Bensen (2017) also supported the role of applying MALL in boosting long-term memory. Furthermore, Cheng and Chen (2022) asserted that MALL provide valuable contextualized language learning. In the same vein, Lei et al. (2022) noted that MALL guarantees learners’ engagement, resulting in a more self-regulated, meaningful, and deeper learning. Similar to the present research’s outcomes, Li and Hafner (2022) as well as Lin and Lin (2019) concluded that implementing MALL enhance vocabulary learning and increase learners’ involvement. Mortazavi et al. (2021) also documented that MALL methods could improve both productive and receptive language skills.

The other possibility for improvement of the experimental group in comparison to the control group in this study lies in the fact that diversity and novelty in the types of proposed tasks on Instagram immerse students in the learning activities (presentation and practice stages). This implication was supported by previous studies, which confirmed the influential role of Instagram as a MALL tool in boosting learners’ motivation, engagement, and attitudes toward learning (Erarslan, 2019; Gonulal, 2019; Klimova and Polakova, 2020; Rasyiid et al., 2021; Guo, 2022). Taking a similar path, Chen Hsieh et al. (2017) provide evidence for the effectiveness of LINE in sustaining an engaging and encouraging language learning atmosphere. From the view point of teachers, Wardak (2021) asserted that MALL facilitate vocabulary development and saving in-class time for other activities. Wrigglesworth (2019) also suggested the usage of smartphones to extend interaction beyond the EFL classroom. In the same vein, Wissam et al. (2020) highlighted the efficiency of social media in second language acquisition and its significant role in developing self-awareness and self-evaluation among the learners.

The finding of this study is in accord with the previous studies, which have shown that applying MALL in learning English through SN tools affects different aspects of Students’ language development positively. For instance, Ramalia (2021) found that Instagram could foster writing assignments. Similarly, Tarigan et al. (2021) confirmed the Instagram platform is an efficient choice for creative story telling among language learners. They also added that language learning via Instagram platform results in academic achievement satisfaction and cooperative learning. Kaviani et al. (2018) also demonstrated that Instagram a useful learning opportunity for language learning among EFL learners. Furthermore, Ngui et al. (2020) provided evidence for the contributions of MALL in autonomous learning. Taking a similar path, Kaviani (2022) used MALL as an instructional medium to teach speaking to EFL Taiwanese students. According to their findings, learners’ immersion in learning activities through MALL could improve their speaking skills. The results of this research are also congruent with Zain et al. (2021) who provided evidence for fostering role of MALL-language learning at university.

As was mentioned before, the intended posts for teaching grammar tips on our Instagram page were designed in pictures, audio, as well as videos. The learners’ positive feedback and immersion in learning activities could suggest that they were more encouraged to listen and watch the explanation of grammatical points through audios and videos. This rationale can be put forward that stimulating learners’ visual-spatial and musical aptitudes promotes active learning. Similar to this research outcome, Alharbi (2019) addressed the issue of video-based grammar teaching and concluded this form of instruction immersed students in active learning. In the same vein, Reynolds and Taylor (2020) utilized the mobile application Kahoot! As an instructional medium to boost vocabulary learning. As they found out, utilizing videos and audio in teaching vocabulary triggers engaging learning.

### Discussing the second research question

Concerning the second research question, the research outcomes suggest that learners’ attitudes toward applying social media to support their learning process were significantly positive. This outcome can be justified due to various traits of social media. For example, one significant quality of Instagram is that it serves both the cognitive and affective needs of educators (Carpenter et al., 2020). Thus, it can be implied that via Instagram, learners are able to acquire knowledge and exchange emotional support. Another offering of social media in general and Instagram, in particular, is enhancing collaborative tasks in a distressful atmosphere. In other words, social media play an essential role in authentic interaction among peers. In this regard, Albadry (2017) and Chang (2020) argued that interactions within social media platforms afford students a rich environment for mastery of language learning. In the same line of inquiry, Lailiyah and Setiyaningsih (2020) concluded that Instagram is the most popular social media. Their findings revealed that learners had positive attitudes toward Instagram provides a new way of learning language. Taking a similar path, Gonulal (2019) asserted that the use of Instagram in language learning encourage learners to be more active.

Furthermore, the efficiency of Instagram feed-based tasks can be attributed to its potential to activate learners’ self-awareness, self-assessment, as well as autonomy. A reasonable explanation is that the implementation of Instagram feed-based tasks provides new dynamics to learners’ self-government. Through Instagram feed-based tasks, learners are afforded to monitor their progress and assume responsibility for their own learning. They can also benefit from their peers’ feedback aside from their self-formulated strategies that assist cooperative learning. This implication is supported by connectivism, which discusses the contributions of social media to self-organization of the learners. This implication is in accord with the findings of Sung et al. (2015) and Muftah (2022), confirming the effectiveness of MALL in improving learners’ autonomy. The other essence of social media is fostering collaborative learning, which is one of the underlying foundations of social-constructivism.

## Conclusion and implications

On the whole, this study was an attempt to contribute to the sparse knowledge on the effects of Instagram feed-based tasks in fostering grammar learning among EFL intermediate learners.

As the findings of the present study indicated, Instagram feed-based tasks are quite beneficial in grammar learning progress in the L2 contexts. Analysis of the result reflected that EFL university professors revealed positive attitudes toward the use of social media, especially Instagram for English grammar learning. They believed that learning through Instagram has the advantage of accelerating Grammar Enrichment Activities. This study opens a new window in teaching and learning grammar and calls for more attention to the role of Instagram feed-based tasks in the L2 learning process.

The findings of this study suggest some pedagogical implications for the learners, teachers, as well as educational system. It is important to note that incorporating MALL into curricula can increase learner access to course content much easily and provide opportunities for interaction with the course outside of the classroom. Moreover, it was approved that MALL is an affordance for learning and practicing grammar in that the learners are engaged to produce or consume content on a readily available device of their choice. One more point which deserves special attention is the unique role of teachers in applying Instagram feed-based tasks in the L2 classrooms. While L2 digital literacy is necessary for practicing social media, it is suggested that L2 teachers should be equipped with knowledge about this influential factor in pre-and in-service teacher education programs. In other words, L2 teachers should be trained in such a way so that they will be able to use social media effectively. Therefore, policy makers, curriculum designers, and teacher educators are asked to consider such training programs for teachers and university professors. In this way, the learning environment will take advantage of more fruitful instruction.

## Limitations and suggestions for future research

The findings of this study should be considered in light of some limitations: Firstly, the demographic information of the learners such as age, gender, and language level were not considered. Thus, it is recommended to consider these factors in similar research in future studies. Furthermore, the relationship between EFL learners’ different socio-cultural backgrounds and Instagram feed-based tasks can be further explored in future studies. It is also suggested to undertake further research on the influence of Instagram feed-based tasks on other skills such as vocabulary expansion, listening, speaking, reading, pronunciation as well as writing. Another limitation of the present study could be attributed to its quasi-experimental design, which used intact groups a sampling procedure. Moreover, a rather small number of participants in both control and experimental groups makes the generalizability of the results limited. Future studies can conduct a similar study with more participants using experimental design. Further studies are required to employ other research approaches such as class observations, dairy, and focused group interviews to investigate the effects of Instagram feed-based tasks in more detail. Additionally, materials developers and curriculum designers are suggested to consider the contributions of MALL-based instruction in designing curriculum, instruction, and materials intended to facilitate language learning. Moreover, the same study is suggested to be conducted in other educational contexts such as schools and private language institutes. Last but not least, exploring the possible similarities and differences between the effects of Instagram feed-based tasks and other social media is recommended for further future studies.

## Data availability statement

The original contributions presented in this study are included in the article/supplementary material, further inquiries can be directed to the corresponding author.

## Author contributions

All authors listed have made a substantial, direct, and intellectual contribution to the work, and approved it for publication.
